# miR-4293 upregulates lncRNA WFDC21P by suppressing mRNA-decapping enzyme 2 to promote lung carcinoma proliferation

**DOI:** 10.1038/s41419-021-04021-y

**Published:** 2021-07-23

**Authors:** Qian Zhang, Yun-Fei Yan, Qing Lv, You-Jie Li, Ran-Ran Wang, Guang-Bin Sun, Li Pan, Jin-Xia Hu, Ning Xie, Can Zhang, Bao-Cheng Tian, Fei Jiao, Sen Xu, Ping-Yu Wang, Shu-Yang Xie

**Affiliations:** 1 Department of Biochemistry and Molecular Biology, Binzhou Medical University, YanTai, ShanDong P. R. China; 2 Department of Chest Surgery, YanTaiShan Hospital, YanTai, ShanDong P. R. China; 3 Genetics and Aging Research Unit, Mass General Institute for Neurodegenerative Diseases (MIND), Department of Neurology, Massachusetts General Hospital and Harvard Medical School, Charlestown, MA USA

**Keywords:** Long non-coding RNAs, Oncogenes

## Abstract

Non-coding RNAs (ncRNAs) involve in diverse biological processes by post-transcriptional regulation of gene expression. Emerging evidence shows that miRNA-4293 plays a significant role in the development of non-small cell lung cancer. However, the oncogenic functions of miR-4293 have not been studied. Our results demonstrated that miR-4293 expression is markedly enhanced in lung carcinoma tissue and cells. Moreover, miR-4293 promotes tumor cell proliferation and metastasis but suppresses apoptosis. Mechanistic investigations identified mRNA-decapping enzyme 2 (DCP2) as a target of miR-4293 and its expression is suppressed by miR-4293. DCP2 can directly or indirectly bind to WFDC21P and downregulates its expression. Consequently, miR-4293 can further promote WFDC21P expression by regulating DCP2. With a positive correlation to miR-4293 expression, WFDC21P also plays an oncogenic role in lung carcinoma. Furthermore, knockdown of WFDC21P results in functional attenuation of miR-4293 on tumor promotion. In vivo xenograft growth is also promoted by both miR-4293 and WFDC21P. Overall, our results establish oncogenic roles for both miR-4293 and WFDC21P and demonstrate that interactions between miRNAs and lncRNAs through DCP2 are important in the regulation of carcinoma pathogenesis. These results provided a valuable theoretical basis for the discovery of lung carcinoma therapeutic targets and diagnostic markers based on miR-4293 and WFDC21P.

## Introduction

Lung carcinoma remains a leading cause of cancer death globally. In spite of advances in early detection and standard treatment, lung cancer is often diagnosed at an advanced stage and has a poor prognosis [1, 2]. The first step in developing effective diagnosis and therapy is to elucidate the molecular mechanism operative in lung carcinoma. Recent large-scale genomic analyses of lung carcinoma identified many potential targets that can be exploited in diagnosis and therapies [3].

Non-coding RNAs (ncRNAs), such as microRNAs (miRNAs) and long non-coding RNAs (lncRNAs), are regulators of intracellular and intercellular signaling in lung cancer and modulate cell signaling to control diverse cellular processes, including proliferation, migration, and apoptosis. Compared to miRNAs, lncRNAs exhibit more wide range of sizes and functions [4]. In tumorigenesis of lung carcinoma, some lncRNAs serve as tumor-suppressive roles such as MEG3 and GAS5 [5, 6], while some are oncogenes like HOTAIR and MALAT1 [7, 8]. As important regulators in gene expression networks, lncRNA can modulate mRNA stability, translation, and post-translation in the cytoplasm. In the nucleus, lncRNAs are capable of controlling nuclear architecture and transcription [9].

Among the versatile function of lncRNA, the crosstalk between lncRNA and miRNA is probably the most reported. As the competing endogenous RNA, lncRNAs can act as miRNA decoys to modulate gene expression [10, 11]. Indeed, miRNAs can also directly or indirectly affect the expression of lncRNAs [12]. In the process of miRNA-mediated gene silencing, miRNA-induced silencing complexes can recruit the cellular mRNA decay machinery to degrade mRNAs and lncRNAs in which decapping complexes are important factors [13, 14]. Components of decapping complexes, including DCP1 and DCP2, can remove 7-methylguanosine (m7G) cap to initiate mRNAs decay [15, 16]. Similarly, decapping complexes also modulate the expression of lncRNAs through RNA degradation, which serves a vital role in transcriptional regulation specifically at inducible genes [17]. The role of lncRNA degradation in the pathogenesis of carcinoma has not been clarified.

MiR-4293 SNP rs12220909 plays a significant role in the development of non-small cell lung cancer (NSCLC) [18–20]. However, the target genes of miR-4293 have not been reported specific function of miR-4293 in NSCLC is unknown. In this study, we presented evidence that DCP2 is a target of miR-4293 and that miR-4293 is highly expressed in lung carcinoma tissue and promotes cell proliferation and migration, but inhibits apoptosis. More importantly, we revealed that the degradation of lncRNA-WFDC21P played important role in the tumorigenesis of lung carcinoma, and demonstrated that by regulating DCP2, miR-4293 can elevate the expression of WFDC21P, which is described as a STAT3 binding lncRNA and specific regulator of human dendritic cell differentiation involved in some immune processes [21, 22]. Here we demonstrate that WFDC21P plays a proto-oncogenic role in lung carcinoma. In addition, miR-4293 and WFDC21P are found possibly participating in a common pathway to regulate STAT3 activation. The oncogenic potential of miR-4293 can be attenuated by blocking WFDC21P. In addition, both miR-4293 and WFDC21P enhance xenograft growth in vivo.

## Results

### MiR-4293 levels significantly increased in lung carcinoma cells and tissues

**Fig. 1:**
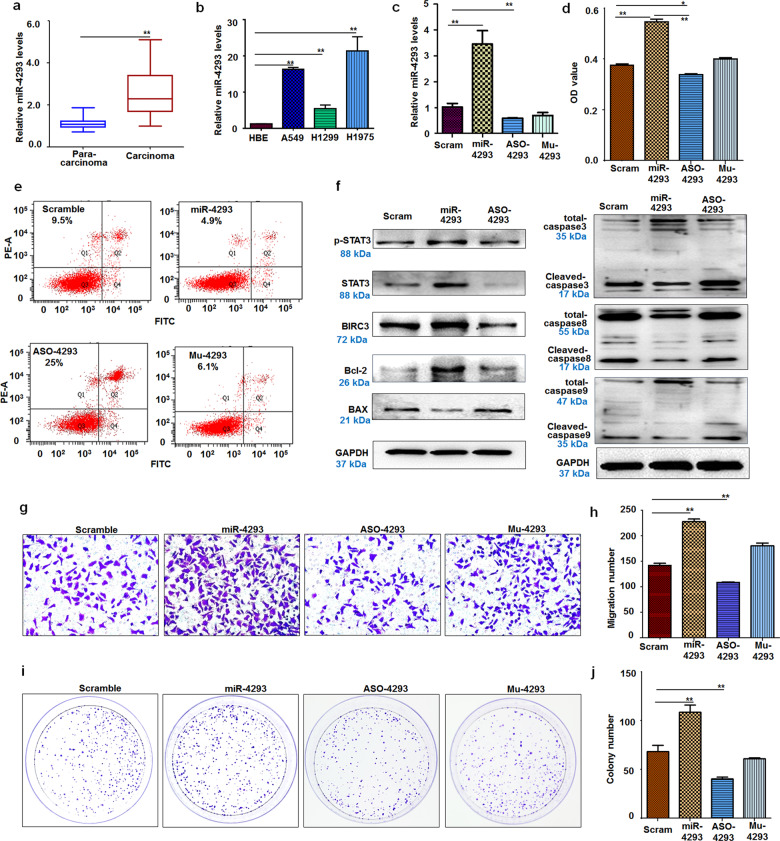
miR-4293 regulates lung cancer cell proliferation, migration, and apoptosis. **a** qRT-PCR analysis of miR-4293 levels in NSCLC tissues and corresponding para-carcinoma tissues (*n* = 16). Data are expressed as median (interquartile range). **P < 0.01; Mann–Whitney U test. **b** qRT-PCR analysis of miR-4293 levels in A549, H1299, H1975, and HBE cells. Data are expressed as mean ± SD for triplicate experiments. **p < 0.01; ANOVA test. **c** qRT-PCR analysis of miR-4293 level in A549 cells with transfection of miR-4293 mimic, ASO-4293, mu-4293 (mutant), or scrambled. Data are expressed as mean ± SD for triplicate experiments. **p < 0.01; ANOVA test. **d** MTT assay of A549 at 24 h post-transfection of miR-4293, ASO-4293, mu-4293 or scrambled. Data are expressed as mean ± SD for triplicate experiments. **p < 0.01, *p < 0.05; ANOVA test. **e** FACS analysis of A549 cell apoptosis at 24 h post-transfection of miR-4293, ASO-4293, mu-4293, or scrambled. **f** Western blot detection of STAT3 and apoptosis-related factors (Bcl-2, Bax, BIRC3, and Caspase 3,8,9) in miR-4293-, ASO-4293-, or scramble-treated A549 cells. **g**, **h** Transwell migration assay of A549 at 24 h post-transfection of miR-4293, ASO-4293, mu-4293, or scrambled. Data are expressed as mean ± SD for triplicate experiments. **p < 0.01; ANOVA test. **i**, **j** Colony formation assays of A549 at 24 h post-transfection of miR-4293, ASO-4293, mu-4293, or scrambled. Data are expressed as mean ± SD for triplicate experiments. **p < 0.01; ANOVA test.

miR-4293 SNP is associated with susceptibility to NSCLC [19], but the molecular function of miR-4293 in NSCLC is still unknown. To further investigate whether miR-4293 is involved in the pathogenesis of lung cancer, we first collected NSCLC sample tissues and corresponding non-tumor tissues. Compared with lung para-carcinoma samples, miR-4293 levels were elevated in lung carcinoma samples (Fig. 1a). Moreover, we also found that the expression of miR-4293 in cancer cell lines (A549, H1299, and H1975) was markedly elevated compared with human bronchial epithelial cells (HBE) (Fig. 1b). These results indicate that miR-4293 may have oncogenic potential in lung carcinoma.

### MiR-4293 promotes cell proliferation and migration

In order to further study the role of miR-4293 in lung carcinoma, we prepared miR-4293 (mimic), ASO-4293 (inhibitor), and mu-4293 (mutant). A549 cells were then transfected with miR-4293, ASO-4293, or Mu-4293 respectively. After transfection, miR-4293 mimic could significantly elevate the miR-4293 level, which could be markedly attenuated by ASO-4293 (Fig. 1c). MTT assays showed that miR-4293 transfection could significantly promote A549 or H1975 cell proliferation compared with cells transfected with scrambled control or ASO-4293. ASO-4293 transfection suppressed cancer cell proliferation compared with the scrambled control (Fig. 1d and Supplemental Fig. 1a). The apoptosis assay showed that miR-4293 treatment decreased lung cancer cell (A549 or H1975) apoptosis compared with the scrambled control. More importantly, miR-4293 treatment significantly increased survival capacity compared with ASO-4293 treatment (Fig. 1e and Supplemental Fig. 1b).

Next, we investigated the effects of miR-4293 on apoptosis-related factors. We found that the expression of anti-apoptosis factors (Bcl2 and BIRC3) could be elevated by miR-4293 transfection, but was attenuated by ASO-4293 treatment compared with scrambled control. However, the expression of Bax, a pro-apoptosis factor was suppressed by miR-4293 treatment and enhanced by ASO-4293 transfection. We also examined the expression of caspases influenced by miR-4293 and ASO-3. miR-4293 treatment resulted in less cleavage of caspase-3, caspase-8, and caspase-9, which play essential roles in apoptosis. Whereas ASO-4293 treatment induced the cleavage of caspase-3, caspase-8, and caspase-9 compared with miR-4293. Importantly, the apoptotic factors examined are closely related to STAT3 signaling pathways; therefore, we detected STAT3 expression and found that miR-4293 treatment could significantly enhance STAT3 phosphorylation compared with a scrambled control, in contrast, ASO-4293 treatment suppressed STAT3 phosphorylation compared with miR-4293 transfection (Fig. 1f and Supplemental Fig. 1c). To investigate the possible role of miR-4293 in tumor cell migration, we conducted Transwell migration assays, which demonstrated that A549 or H1975 cells transfected with miR-4293 have enhanced migration capacity compared with cells transfected with scrambled control (Fig. 1g, h, and Supplemental Fig. 1d, e). Additionally, miR-4293 treatment could significantly increase the survival of A549 or H1975 cells seeded sparsely in a clonogenic assay compared with the scrambled control. Conversely, cells transfected with ASO-4293 formed fewer colonies compared with scrambled control (Fig. 1i, j and Supplemental Fig. 1f, g). miR-4293 promoted cell proliferation and migration but inhibited apoptosis, which was also confirmed in H1299 cells (Supplemental Fig. 2) These results indicate that miR-4293 plays a significant role in lung carcinoma pathogenesis.

**Fig. 2:**
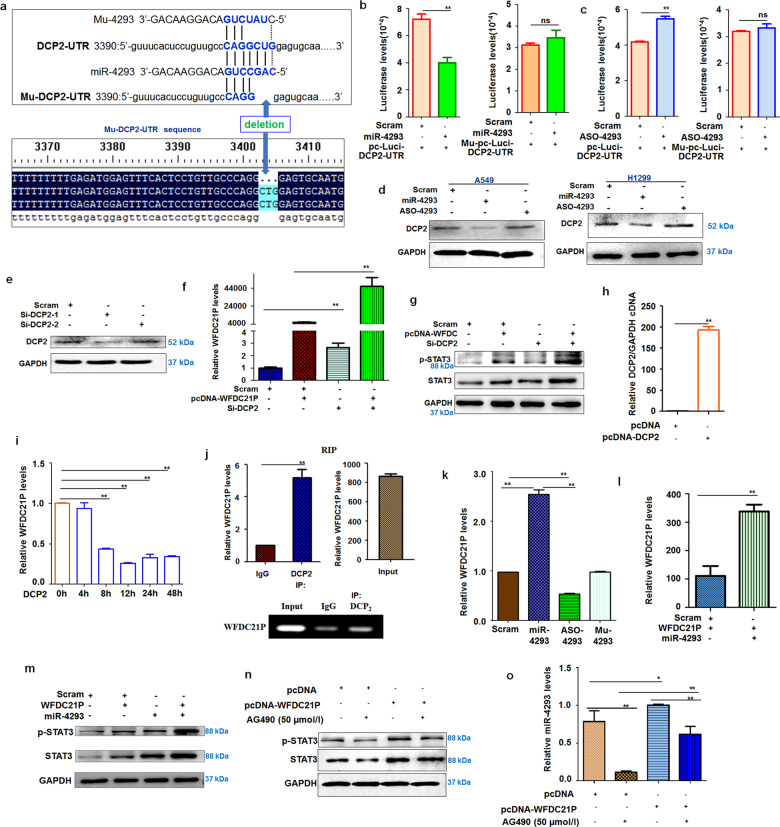
DCP is targeted by WFDC21P, which is elevated by miR-4293. **a** TargetScan analysis predicted that the DCP2 mRNA-3′-UTR is targeted by miR-4293. We synthesized a mutant miR-4293 (Mu-4293) and cloned DCP2 mRNA-3’-UTR and mu-DCP2–3’-UTR sequences. **b** Analysis of miR-4293 regulating luciferase expression of reporter plasmids containing DC2–3’UTR (right panel) or mu-DCP2–3’UTR (left panel). Data are expressed as mean ± SD for triplicate experiments. **p < 0.01; Student’s *t*-test. **c** Analysis of ASO-4293 regulating luciferase expression of reporter plasmids containing DC2–3’UTR (right panel) or mu-DCP2–3’UTR (left panel). Data are expressed as mean ± SD for triplicate experiments. **p < 0.01; Student’s *t*-test. **d** Western blot detection of DCP2 in miR-4293-, ASO-4293-, or scramble-treated A549 cells. **e** Western blot detection of DCP2 in siRNA-DCP2–1-, siRNA-DCP2–2-, or scramble-treated A549 cells and H129 cells. **f** qRT-PCR analysis of WFDC21P level in pcDNA-WFDC21P + scramble-, si-DCP2-, si-DCP2 + pcDNA-WFDC21P-, or scramble-treated A549 cells. Data are expressed as mean ± SD for triplicate experiments. **p < 0.01; ANOVA test. **g** Western blot detection of STAT3 phosphorylation in pcDNA-WFDC21P + scramble-, si-DCP2-, si-DCP2 + pcDNA-WFDC21P-, or scramble-treated A549 cells. **h** qRT-PCR analysis of DCP2 level in pcDNA or pcDNA-DCP2 -treated A549 cells. Data are expressed as mean ± SD for triplicate experiments. **p < 0.01; Student’s *t*-test. **i** qRT-PCR analysis of WFDC21P level in 0 h, 4 h, 8 h, 12 h, 24 h, 48 h after transfection of pcDNA-DCP2. Data are expressed as mean ± SD for triplicate experiments. **p < 0.01; ANOVA test. **j** RIP assay of the interaction between WFDC21P and DCP2. Upper panel, qRT-PCR analysis of WFDC21P pull-down by IgG or DCP2; lower panel, electrophoresis for WFDC21P pull-down by IgG or DCP2 after PCR amplification. Data are expressed as mean ± SD for triplicate experiments. **p < 0.01; Student’s *t*-test. **k** qRT-PCR analysis of WFDC21P levels in miR-4293-, ASO-4293, Mu-4293 or scramble-treated A549 cells. Data are expressed as mean ± SD for triplicate experiments. **p < 0.01; ANOVA test. **l** qRT-PCR analysis of WFDC21P levels in miR-4293+pcDNA-WFDC21P-, or scramble+ pcDNA-WFDC21P-treated A549 cells. Data are expressed as mean ± SD for triplicate experiments. **p < 0.01; Student’s *t*-test. **m** Western blot detection of STAT3 phosphorylation in pcDNA-WFDC21P + scramble-, miR-4293-, miR-4293+pcDNA-WFDC21P-, or scramble-treated A549 cells. **n** Western blot detection of STAT3 phosphorylation in AG490 + pcDNA-, pcDNA-WFDC21P-, AG490 + pcDNA-WFDC21P-, or pcDNA treated A549 cells. **o** qRT-PCR analysis of miR-4293 level in AG490 + pcDNA-, pcDNA-WFDC21P-, AG490 + pcDNA-WFDC21P-, or pcDNA treated A549 cells. Data are expressed as mean ± SD for triplicate experiments. **p < 0.01, *P < 0.05; ANOVA test.

### MiR-4293 targets and suppresses DCP2 expression

It is well known that miRNAs can induce gene silencing by binding the 3′ untranslated region of targeted mRNAs. To further study the tumor promotion role of miR-4293, we used miRNA TargetScan (http://www.targetscan.org/vert_72/) to predict the mRNAs that may bind to miR-4293. This analysis indicated that DCP2, a key component for degrading RNA, may be a target of miR-4293 (Fig. 2a). A luciferase reporter plasmid containing DCP2–3′UTR was then constructed and used to co-transfect cells with miR-4293. miR-4293 transfection significantly decreased the expression of luciferase. To further confirm that miR-4293 binds to the DCP2–3′UTR sequence, we constructed a mu-DCP2–3′UTR luciferase reporter plasmid in which we deleted the “CTG” sequence from the DCP2–3′UTR luciferase reporter plasmid. As a result, cells, co-transfected with mu-DCP2–3′UTR and miR-4293, had no significant downregulation of luciferase (Fig. 2b). As expected, miR-4293 inhibitor (ASO-4293) co-transfected with DCP2–3′UTR luciferase reporter plasmid elevated the expression of luciferase, while luciferase of mu-DCP2–3′UTR luciferase reporter plasmid could not be upregulated by ASO-4293 (Fig. 2c). Additionally, we also constructed GFP reporter plasmids containing DCP2–3′UTR or mu-DCP2–3′UTR. co-transfected with DCP2–3′UTR GFP reporter plasmid, miR-4293 resulted in considerable attenuation of both GFP intensity and the percentage of GFP-positive cells. While, miR-4293 could not decrease the GFP intensity or the percentage of GFP-positive cells with co-transfection of mu-DCP2–3′UTR. Cells were then co-transfected with mu-4293 (containing a mutated miR-4293 binding site) and the DCP2–3′-UTR GFP reporter plasmid. mu-4293 could not reduce the percentage of GFP-positive cells compared with the wide type miR-4293 (Supplemental Fig. 3a). Next, we transfected cancer cells with miR-4293 and its inhibitor (ASO-4293). In accordance with the results of reporter experiments, miR-4293 transfection significantly attenuated DCP2 expression compared to transfection of both ASO-4293 and scrambled control (Fig. 2d). These results indicate that miR-4293 can downregulate the expression of DCP2.

**Fig. 3:**
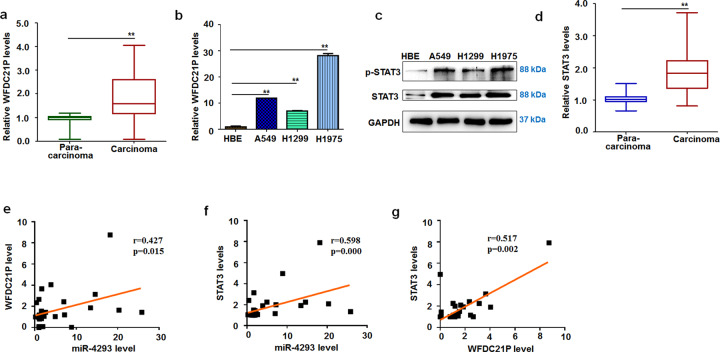
WFDC21P and STAT3 levels are increased in NSCLC. **a** qRT-PCR analysis for WFDC21P level in NSCLC tissues and corresponding para-carcinoma tissues (*n* = 16). Data are expressed as median (interquartile range). **P < 0.01; Mann–Whitney U test. **b** qRT-PCR analysis for WFDC21P level in A549, H1299, H1975, and HBE cells. Data are expressed as mean ± SD for triplicate experiments. **p < 0.01; ANOVA test. **c** Western blot detection of STAT3 in A549, H1299, H1975, and HBE cells. **d** qRT-PCR analysis of STAT3 expression in NSCLC tissues and corresponding para-carcinoma tissues (*n* = 16). Data are expressed as median (interquartile range). **P < 0.01; Mann–Whitney U test. **e**–**g** Pearson′s correlation analysis of miR-4293 expression and WFDC21P expression (*n* = 16, **P = 0.015) (**e**), miR-4293 expression and STAT3 expression (n = 16, **P < 0.01) (**f**), WFDC21P expression and STAT3 expression (*n* = 16, **P < 0.01) (**g**).

### DCP2 binds and regulates WFDC21P levels

DCP2 is involved in the pathogenesis of some carcinoma by degrading RNA [23]. According to the above results, miR-4293 can downregulate the expression of DCP2, which is able to promote the degradation of long noncoding RNA [24–26]. Our results also demonstrated that miR-4293 can significantly enhance STAT3 phosphorylation. In addition, STAT3 phosphorylation can be elevated by WFDC21P in human dendritic cell differentiation [21, 22]. Therefore, we assumed that WFDC21P may be regulated by DCP2, and is involved in the pathogenesis of lung cancer. To investigate this hypothesis, we first screened for an effective siRNA against DCP2 (Fig. 2e).

Then, we detected the role of DCP2 in regulating WFDC21P expression. As expected, pcDNA-WFDC21P transfection elevated WFDC21P levels compared with the scrambled control. After DCP2 knockdown, irrespective of whether WFDC21P was overexpressed or not, WFDC21P was significantly elevated compared with the scrambled control. More importantly, DCP2 knockdown combined with pcDNA-WFDC21P transfection resulted in further increased WFDC21P expression compared with control treatment (pcDNA-WFDC21P + Scram) (Fig. 2f). As a regulated target of WFDC21P, STAT3 phosphorylation was also used to evaluate the effects of DCP2 on WFDC21P. As expected, WFDC21P overexpression after attenuating DCP2 by siRNA could further enhance STAT3 phosphorylation compared with scrambled control treatment. STAT3 phosphorylation was also higher in cells co-transfected with si-DCP2 and pcDNA-WFDC21P compared with control treatment (Fig. 2g). To investigate the underlying mechanisms that DCP2 downregulated the expression of WFDC21P, DCP2 overexpression plasmid (pcDNA-DCP2) was constructed and transfected into the A549 cells, then the expression of WFDC21P was detected over a time course. As a result, there was no major change in the expression of WFDC21P during pcDNA-DCP2 transfection 0–4 h. Interestingly, the expression of WFDC21P was significantly attenuated at 8 h after pcDNA-DCP2 transfection (Fig. 2h, i), while pcDNA3.1 transfection could not affect the expression of WFDC21P during the whole 48 h (Supplemental Fig. 3b). Furthermore, DCP2 can bind to RNA and promote its degradation by decapping [24]. Therefore, RNA immunoprecipitation (RIP) was performed to check the interaction between WFDC21P and DCP2. As a result, WFDC21P was significantly enriched by DCP2 immunoprecipitation compared with control IgG precipitation, indicating that WFDC21P interacts with DCP2 directly or indirectly (Fig. 2j). These results suggest that DCP2 downregulates the expression of WFDC21P.

### MiR-4293 elevates WFDC21P levels and STAT3 activation by suppressing DCP2

The above results indicate that WFDC21P is downregulated by interaction with DCP2 and that DCP2 expression is suppressed by miR-4293. However, the effects of miR-4293 on WFDC21P were still to be elucidated. Firstly, we found that miR-4293 treatment could markedly elevate WFDC21P levels compared with the scrambled control. In contrast, the ASO-4293 treatment suppressed WFDC21P expression compared with the scrambled control (Fig. 2k). In addition, miR-4293 treatment further increased the WFDC21P level (Fig. 2l). These results indicate that miR-4293 elevation can induce the expression of WFDC21P.

Next, STAT3 activation was used to evaluate the relationship between WFDC21P and miR-4293. As expected, overexpression of both WFDC21P and miR-4293 resulted in more STAT3 phosphorylation compared with only WFDC21P or miR-4293 treatment (Fig. 2m). To further investigate the role of WFDC21P in stimulating the STAT3 pathway, we treated WFDC21P-overexpressed cells with AG490 (a specific inhibitor to STAT3 signaling, 50μmol/L), and found that AG490 treatment reduced WFDC21P-induced STAT3 activation (Fig. 2n).

We also found that WFDC21P, by regulating the STAT3 pathway, may have a positive feedback effect on the expression of miR-4293. STAT3 pathway was deactivated by AG490 treatment, which resulting in the decreased levels of miR-4293. Moreover, miR-4293 elevation resulting from WFDC21P overexpression was further alleviated by using AG490 to deactivate the STAT3 pathway (Fig. 2o). These results indicate that there might be a feedback loop between miR-4293 and WFDC21P in which miR-4293 increases WFDC21P levels and WFDC21P might upregulate miR-4293 expression by activation of STAT3 pathways.

### WFDC21P and STAT3 levels increased in NSCLC cells and tissues

The above results indicate that WFDC21P expression and STAT3 activation can be upregulated by miR-4293. However, the role of WFDC21P and STAT3 in the pathogenesis of NSCLC requires clarification. Firstly, carcinoma tissue lysates prepared from 3 NSCLC patients and controls from their corresponding para-carcinoma tissues were subjected to RNA-seq analysis to detect mRNA expression. Mining of the RNA-seq dataset identified that the expression of 1392 mRNAs was elevated, and 1303 were downregulated (fold change > 2.0, *p* < 0.05) (Supplemental Fig. 4a). Accumulated evidence indicated that the WFDC family played great roles in the progression of carcinoma. [27–29] Additionally, some of them have been identified as biomarkers of diagnosis of lung cancer. Here, the members of the WFDC family were selected to do a further comprehensive analysis of their roles in lung cancer. Our results demonstrated that WFDC1 was attenuated while WFDC2 was significantly elevated in carcinoma tissue. As a member of the WFDC family, we also found that lncRNA-WFDC21P may have enhanced expression in carcinoma tissue (Supplemental Fig. 4b). Moreover, the role of WFDC21P in tumorigenesis of lung cancer is unclear till now. Therefore, we collected more NSCLC sample tissues and corresponding non-tumor tissues to further investigate the WFDC21P expression in lung cancer. Compared with lung para-carcinoma samples, WFDC21P levels were elevated in lung carcinoma samples (Fig. 3a). Additionally, we also found that the expression of WFDC21P in cancer cell lines (A549, H1299, and H1975) was markedly elevated compared with human bronchial epithelial cells (HBE) (Fig. 3b). As a direct target of WFDC21P, STAT3 phosphorylation was increased in lung carcinoma cells (Fig. 3c). Similarly, STAT3 expression was also increased in lung carcinoma tissue (Fig. 3d). Based on the above results, we further analyzed possible correlations among WFDC21P, STAT3, and miR-4293 in the NSCLC samples. We found that miR-4293 expression was positively correlated with WFDC21P expression (*p* = 0.015, Fig. 3e). miR-4293 and STAT3 expression was also in positive correlation (*p* < 0.01, Fig. 3f), as was the relationship between WFDC21P and STAT3 expression (*p* < 0.01, Fig. 3g). These results indicate that WFDC21P also has oncogenic potential.

**Fig. 4:**
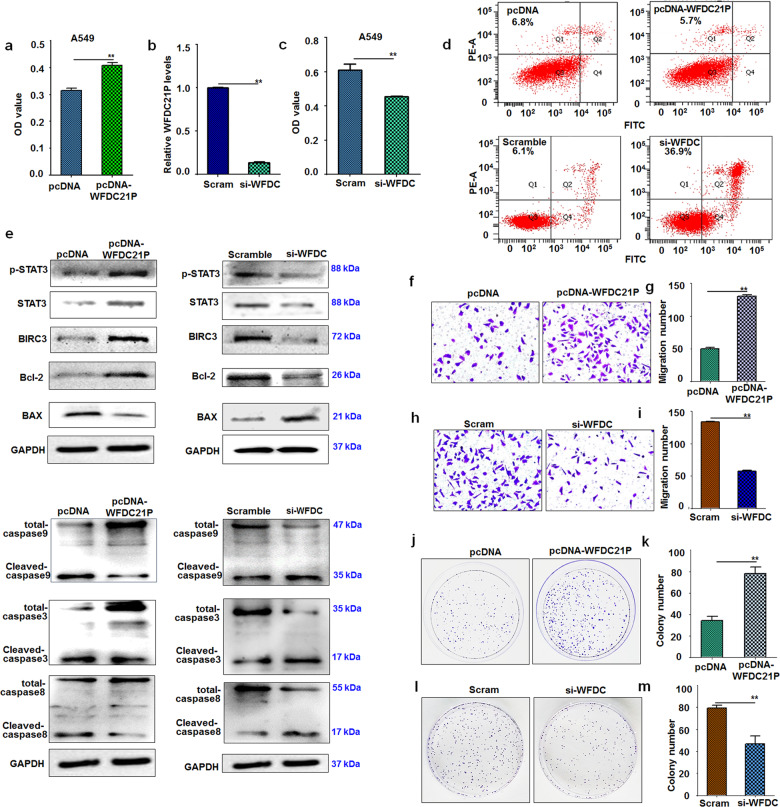
WFDC21P regulates cell proliferation, migration, and apoptosis. **a** MTT assays of A549 at 24 h post-transfection of pcDNA-WFDC21P and pc-DNA. Data are expressed as mean ± SD for triplicate experiments. **p < 0.01; Student’s *t*-test. **b** qRT-PCR analysis of WFDC21P level in si-WFDC21P- and scramble-treated cells. Data are expressed as mean ± SD for triplicate experiments. **p < 0.01; Student’s *t*-test. **c** MTT assays of A549 at 24 h post-transfection of si-WFDC21P and scramble. Data are expressed as mean ± SD for triplicate experiments. **p < 0.01; Student’s *t*-test. **d** FACS analysis of A549 cell apoptosis at 24 h post-transfection of pc-DNA, pcDNA-WFDC21P, si-WFDC21P, and scrambled control. **e** Western blot detection of STAT3 and apoptosis-related factors (Bcl-2, Bax, BIRC3, and Caspase 3,8,9) pcDNA *vs* pcDNA-WFDC21P; scramble *vs* si-WFDC. **f**–**i** Transwell assays of A549 migrat**i**on. **f**, **g** pcDNA *vs* pcDNA-WFDC21P; (**h**) and (**i**) scramble vs. si-WFDC. Data are expressed as mean ± SD for triplicate experiments. **p < 0.01; Student’s *t*-test. **j**–**m** Colony formation assays of A549. **j**, **k** pcDNA *vs* pcDNA-WFDC21P; (**l**) and (**m**) scramble vs. si-WFDC. Data are expressed as mean ± SD for triplicate experiments. **p < 0.01; Student’s *t*-test.

### WFDC21P enhances cell proliferation and migration

To further investigate the role of WFDC21P in the pathogenesis of NSCLC, we examined the effects of WFDC21P on cell proliferation, migration, and apoptosis. WFDC21P overexpression markedly enhanced A549 or H1975 cell growth (Fig. 4a and Supplemental Fig. 5a), while transfection with an effective si-WFDC21P inhibited cell proliferation (Fig. 4b, c and Supplemental Fig. 5b). The role of WFDC21P in apoptosis was also examined, and WFDC21P knockdown increased apoptosis (Fig. 4d and Supplemental Fig. 5c). The expression of apoptotic factors related to STAT3 was examined. Expression of Bcl2 and BIRC3 was significantly attenuated in si-WFDC21P-treated cells, but was elevated by WFDC21P overexpression. WFDC21P knockdown resulted in Bax elevation, while WFDC21P overexpression decreased Bax levels. Additionally, WFDC21P knockdown resulted in higher levels of cleaved caspases (caspase-3, 8, and 9). As expected, WFDC21P overexpression suppressed the cleavage of caspases. The effects of WFDC21P on the examined apoptosis factors in A549 or H1975 cells may be owned to the enhanced phosphorylation of STAT3 resulted from WFDC21P elevation (Fig. 4e and Supplemental Fig. 5d). Therefore, we next examined the effects of WFDC21P on tumor cell migration. WFDC21P overexpression increased the number of cells penetrating the Transwell membrane. Conversely, WFDC21P knockdown attenuated the cell penetration (Fig. 4f–i and Supplemental Fig. 5e–h). In the colony formation assay, overexpression of WFDC21P resulted in an elevation of colony number. As expected, WFDC21P knockdown decreased the number of colonies (Fig. 4j–m and Supplemental Fig. 5i–l). In H1299 cells, we also found WFDC21P could promote cell proliferation and migration (Supplemental Fig. 6). These results indicate that WFDC21P is a proto-oncogene in lung carcinoma.

**Fig. 5:**
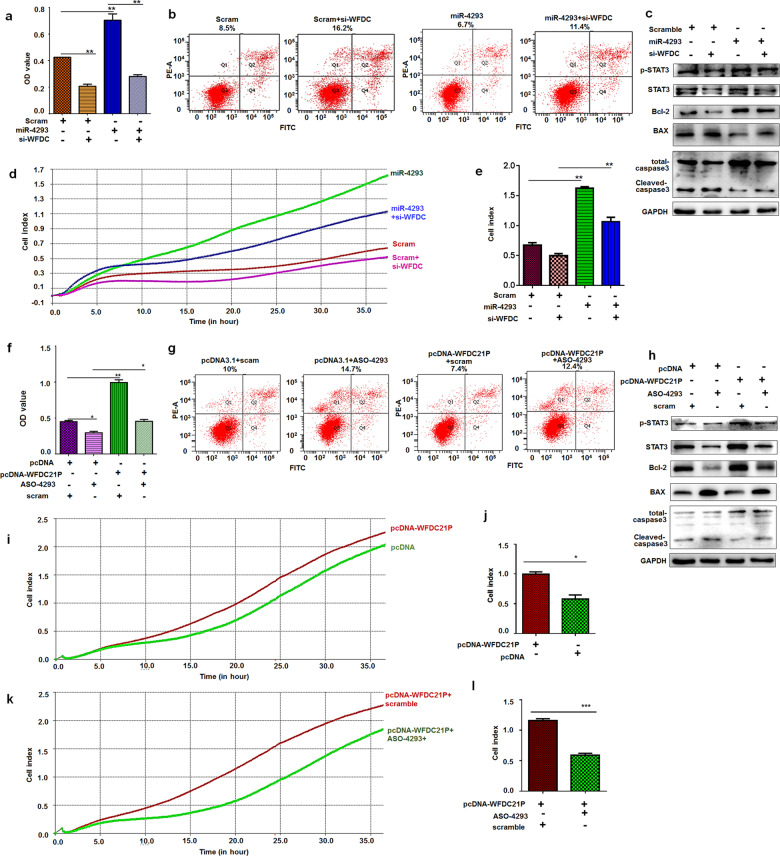
miR-4293 function was attenuated by blocking WFDC21P. The above-mentioned data showed that miR-4293 transfection elevated miR-4293 levels, which could be attenuated by ASO. pcDNA-WFDC21P transfection increased WFDC21P. Si-WFDC treatment reduced WFDC21P level. **a** MTT assay of A549 at 24 h post-transfection of si-WFDC + scramble, miR-4293, miR-4293+si-WFDC, and scramble. Data are expressed as mean ± SD for triplicate experiments. **p < 0.01; ANOVA test. **b** FACS analysis of A549 cell apoptosis at 24 h post-transfection of si-WFDC + scramble, miR-4293, miR-4293+si-WFDC, and scramble. **c** Western blot detection p-STAT3, Bcl-2, BAX, cleaved-caspase 3, 8, and 9 in si-WFDC + scramble-, miR-4293-, miR-4293+si-WFDC- or scramble-treated cells. **d**, **e** RTCA station analysis of migration for A549 with transcription of si-WFDC + scramble-, miR-4293-, miR-4293+si-WFDC- or scramble. **f** MTT of A549 at 24 h post-transfection of pcDNA+ASO-4293, pcDNA-WFDC21P + scramble, pcDNA-WFDC21P + ASO-4293, and pcDNA+ scramble. Data are expressed as mean ± SD for triplicate experiments. (* *P* < 0.05). **g** FACS analysis of A549 cell apoptosis at 24 h post-transfection of pcDNA+ASO-4293, pcDNA-WFDC21P + scramble, pcDNA-WFDC21P + ASO-4293, and pcDNA+ scramble. **h** Western blot detection of STAT3 phosphorylation and apoptosis-related factors (Bcl-2, Bax and Caspase 3) in pcDNA+ASO-4293, pcDNA-WFDC21P + scramble, pcDNA-WFDC21P + ASO-4293, and pcDNA+ scramble-treated A549 cells. **i**–**l** RTCA station analysis of migration for A549. **i**, **j** pcDNA-WFDC21P *vs* pc-DNA; (**k**) and (**l**), pcDNA-WFDC21P + scramble vs. pcDNA-WFDC21P + ASO-4293. Data are expressed as mean ± SD for triplicate experiments. *p < 0.05, ***p < 0.001; Student’s *t*-test.

**Fig. 6:**
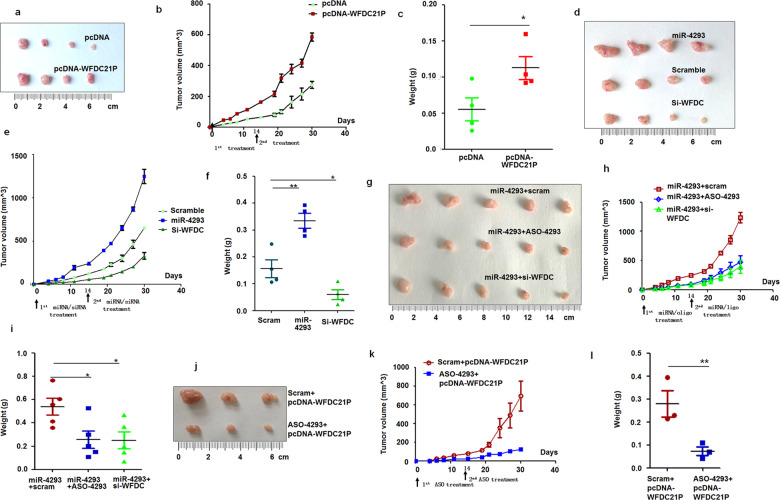
miR-4293 and WFDC21P promote tumorigenesis in vivo. miR-4293 transfection elevated miR-4293 level, which could be reduced by ASO. pcDNA-WFDC21P increased WFDC21P and Si-WFDC treatment reduced WFDC21P level. **a**–**c** Analysis of xenograft of A549 cells treated with pc-DNA or pcDNA-WFDC21P (*n* = 4). Quantitative analysis of tumor volume (**b**) and tumor weight (**c**) of xenografts. The xenografts of each group were weighed and data were expressed as median (interquartile range). *p < 0.05; Mann-Whitney U *t*-test. **d**–**f** Analysis of xenograft tumors of miR-4293-, si-WFDC-, and scramble-treated A549 cells (*n* = 4). Quantitative analysis of tumor volume (**e**) and tumor weight (**f**) of xenografts. Data are expressed as median (interquartile range). **p < 0.01, *p < 0.05; Kruskal-Wallis H test. **g**–**i** Analysis of xenograft tumors of miR-4293-, miR-4293+ASO-4293- and miR-4293+WFDC-treated A549 cells (*n* = 5). Quantitative analysis of tumor volume (**h**) and tumor weight (**i**) of xenografts. Data are expressed as median (interquartile range). **p < 0.01, *p < 0.05; Kruskal-Wallis H test. **j**–**l** Analysis of xenograft tumors of A549 cells treated with pcDNA-WFDC21P + ASO-4293 or pcDNA-WFDC21P + scramble (*n* = 3). Quantitative analysis of tumor volume (**k**) and tumor weight (**l**) of xenografts. Data are expressed as median (interquartile range). **p < 0.01; Mann-Whitney U *t-*test.

### Knockdown of WFDC21P attenuates the oncogenic role of miR-4293

The above results suggest that both miR-4293 and WFDC21P exert an oncogenic role in the proliferation and migration of lung carcinoma cells. Moreover, miR-4293 could promote WFDC21P expression by regulating DCP2. To better understand whether the role of miR-4293 can be attenuated by WFDC21P knockdown, we first evaluated cell proliferation co-influenced by miR-4293 and si-WFDC21P. WFDC21P knockdown suppressed cell proliferation and miR-4293 transfection promoted cell proliferation. More importantly, the increase in cell proliferation resulting from miR-4293 transfection was markedly alleviated by si-WFDC21P (Fig. 5a). The apoptosis assay showed that WFDC21P knockdown promoted apoptosis and also significantly abolished the increased cell viability resulting from miR-4293 transfection (Fig. 5b).

We then examined the expression of Bcl2, anit-apoptosis factors, which was enhanced by miR-4293 transfection and suppressed by WFDC21P knockdown compared with scrambled control. Moreover, Bcl2 elevation caused by miR-4293 treatment was attenuated by WFDC21P knockdown. Besides, miR-4293 transfection with WFDC21P knockdown resulted in lower expression of Bcl2 compared with miR-4293 transfection with scrambled control. The expression of Bax was elevated by WFDC21P knockdown but was suppressed by miR-4293 transfection compared with scrambled control. si-WFDC21P alleviated the regulation of Bax expression affected by miR-4293. Accordingly, WFDC21P knockdown induced more caspase-3 cleaved than miR-4293 treatment. miR-4293 transfection attenuated the elevation of cleavage caspase-3 induced by WFDC21P knockdown. More importantly, we found that miR-4293 transfection enhanced STAT3 phosphorylation, which was alleviated by si-WFDC21P (Fig. 5c). RTCA station analysis was performed to continuously observe cells migration, which gradually increased in miR-4293-treated cells but was blocked by si-WFDC21P treatment (Fig. 5d, e).

To further study whether the oncogenic role of miR-4293 can be attenuated by blocking WFDC21P, we investigated whether ASO-4293 inhibition of lung cancer cell proliferation can be restored by WFDC21P overexpression. We found that the proliferative capacity was suppressed by ASO-4293 and enhanced by WFDC21P overexpression compared with control. More interestingly, the inhibition of cell proliferation by ASO-4293 was restored to a certain extent after WFDC21P overexpression (Fig. 5f). Compared with the control treatment, ASO-4293 transfection increased apoptosis. Even though WFDC21P overexpression could promote cell viability, the percentage of apoptotic cells was still elevated by ASO-4293 transfection (Fig. 5g). By examination of apoptosis-related factors, we found that the expression of Bcl2 was decreased in ASO-4293-transfected cells, but increased in WFDC21P overexpression cells. ASO-transfection with WFDC21P overexpressing resulted in attenuation of Bcl2 expression compared with control treatment. In contrast, Bax expression was significantly enhanced by ASO-4293 transfection. WFDC21P overexpression partially attenuated Bax expression induced by ASO-4293. As expected, WFDC21P overexpression decreased the cleavage of caspase-3 compared with scramble control. And attenuation of caspase-3 cleavage caused by WFDC21P overexpression was alleviated by ASO treatment. We also found that ASO-4293 resulted in less activated STAT3, while WFDC21P overexpression increased STAT3 activation. WFDC21P overexpression also partially increased STAT3 levels induced by ASO-4293 (Fig. 5h). As expected, ASO-4293 treatment could also alleviate migration capacity by WFDC21P overexpression (Fig. 5i-l). Together, these results indicate that the oncogenic activity of miR-4293 can be attenuated by blocking WFDC21P expression.

### MiR-4293 and WFDC21P promote tumorigenesis in vivo

Both miR-4293 and WFDC21P have oncogenic activities in NSCLC. To further investigate the roles of miR-4293 and WFDC21P in vivo, we hypodermically injected A549 cells stably treated with a plasmid, or miRNA, or siRNA, or controls into nude mice to produce xenografts. Every 3 days, we measured the volumes of the xenografts. One month after injection, the mice were sacrificed. Stable expression of WFDC21P resulted in enhanced tumor growth both in volumes and weights compared with pcDNA control (Fig. 6a–c). We examined the expression of WFDC21P and STAT3 phosphorylation in the xenografts. As expected, WFDC21P overexpression in injected xenografts resulted in an elevation of WFDC21P and STAT3 phosphorylation (Supplemental Fig. 7a, b).

**Fig. 7:**
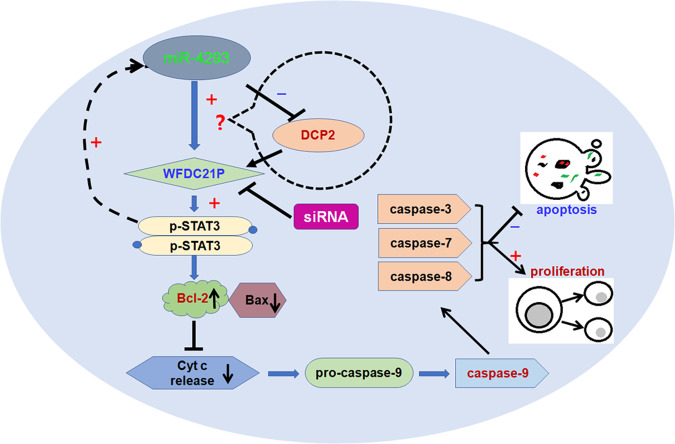
miR-4293 regulating WFDC21P through DCP-2. miR-4293 promotes tumor cell proliferation and metastasis but suppresses apoptosis. DCP2 can directly or indirectly bind to and downregulate WFDC21P. By targeting DCP2 mRNA, miR-4293 suppresses the expression of DCP2, and further enhances the expression of WFDC21P and promotes STAT3 phosphorylation. As a result of elevated STAT3 activity, apoptosis is suppressed and proliferation is enhanced through Bcl2 elevation, Bax2 attenuation and reduced caspase cleavage. In addition, WFDC21P elevates miR-4293 expression by promoting STAT3 activation.

We then evaluated the effects of miR-4293 or WFDC21P knockdown on xenograft growth. miR-4293 treatment enhanced growth in both volumes and weights of xenografts compared with scrambled control. In contrast, WFDC21P knockdown resulted in xenografts growth that was significantly lower than that produced by miR-4293 overexpression and also than scrambled control (Fig. 6d–f). Compared with a scrambled control, miR-4293 treatment resulted in an elevation of miR-4293, WFDC21P, and STAT3 phosphorylation in xenografts. Conversely, these measures were attenuated by WFDC21P knockdown (Supplemental Fig. 7c–e).

To better understand the cooperative action of miR-4293 and WFDC21P in tumor growth, we established xenografts with A549 cells co-treated with miR-4293 and scrambled control, miR-4293 and ASO-4293, as well as miR-4293 and siRNA-WFDC21P. As expected, xenografts with miR-4293 and scrambled control co-treatment had the highest growth rate of these three groups. Interestingly, ASO-4293 and si-WFDC21P could abolish the tumor-promoting effect of miR-4293 almost equally (Fig. 6g–i). Moreover, ASO-4293 attenuated the decrease in WFDC21P or STAT3 levels in xenografts by miR-4293 and si-WFDC21P (Supplemental Fig. 7f–h). Similarly, tumor promotion resulting from WFDC21P stable overexpression was also alleviated by ASO-4293 treatment (Fig. 6j–l). ASO-4293 treatment could also alleviate elevation of WFDC21P and STAT3 phosphorylation in xenografts resulting from WFDC21P overexpression in injected cells (Supplemental Fig. 7i, j). The above results support the ability of miR-4293 and WFDC21P to promote tumor growth in vivo.

## Discussion

miRNAs are involved in cell proliferation, metastasis, and apoptosis, and can also play oncogenic or tumor-suppressive roles [30]. Here, we show that miR-4293 is significantly elevated in NSCLC tissues and promotes tumor cell proliferation and metastasis but suppresses apoptosis. By targeting DCP2 mRNA, miR-4293 suppresses the expression of DCP2. More interestingly, DCP2 can directly or indirectly bind to and downregulate WFDC21P. Moreover, STAT3 is a target gene of WFDC21P [22]. Importantly, our results confirm that miR-4293 enhances the expression of WFDC21P and promotes STAT3 phosphorylation by a novel mechanism in regulating DCP2. In addition, WFDC21P elevates miR-4293 expression by promoting STAT3 activation. As a result of elevated STAT3 activity, apoptosis is suppressed and proliferation is enhanced through Bcl2 elevation, Bax2 attenuation, and reduced caspase cleavage (Fig. 7). Together, these findings provide evidence that miR-4293 plays an oncogenic role in NSCLC by suppressing DCP2-mediated WFDC21P degradation.

Accumulated studies have indicated that the prominent class of lncRNA share a common function in negatively impacting miRNA-mediated gene regulation [31]. Interestingly, we found that in contrast to ceRNA, not only WFDC21P could be up-regulated by miR-4293, but miR-4293 expression could be enhanced by WFDC21P through positive feedback. Both miR-4293 and WFDC21P play oncogenic roles in NSCLC. To better understand the mechanism, a key step is to determine miR-4293 targets and we revealed DCP2 to be such a target. As a critical component of the decapping complex, DCP2 is responsible for RNA degradation [24, 25]. By decapping a subset of mRNAs, DCP2 plays an important role in cancer pathogenesis by affecting processes such as cell migration and apoptosis [26, 32–35]. More interestingly, DCP2 is regulated at the post-transcriptional level rather than being transcriptionally silenced, and miRNAs are an effective way to downregulate DCP2. DCP2 mRNA has a fairly long 3′-UTR of 7.2 kb that may contain many potential miRNA binding sites. miR-141-3p/200a-3p may be involved in the repression of DCP2 expression during renal development [36]; therefore, miRNAs can not only suppress the expression of target genes by binding the 3′-UTR but can also regulate gene expression by involving DCP2 activity. As a novel finding, we observed that miR-4293 can participate in the binding of DCP2 transcripts and downregulate their expression. Several studies indicate that DCP2 appears to target distinct transcripts and the reported targets are mRNAs or lncRNAs [26, 37, 38]. More interestingly, the degradation of lncRNAs mediated by DCP2 serves a vital role in transcriptional regulation specifically at inducible genes [17]. Accordingly, we observed that DCP2 can bind to WFDC21P, a long non-coding RNA, to participate in cancer pathogenesis. We further clarified that WFDC21P downregulation played important role in the tumorigenesis of lung carcinoma, and showed that DCP2 regulated by miR-4293, downregulated WFDC21P expression. The downregulation of lncRNAs mediated by DCP2 may become a novel underlying mechanism of lncRNAs in different kinds of carcinoma. However, DCP2 is not the only one component of the decapping enzyme [39], and the specific underlying mechanism for DCP2 promoting WFDC21P degradation needs further study.

Although we did not confirm a direct interaction between miR-4293 and WFDC21P, miR-4293 can still elevate WFDC21P expression. More importantly, miR-4293 upregulated of WFDC21P in both NSCLC cells and xenografts. In addition, the expression of miR-4293 was positively correlated with WFDC21P levels in lung carcinoma. Hence, the elevation of miR-4293 in NSCLC tissues might account for the increase in WFDC21P levels. Recent studies reveal that lncRNAs, as competing endogenous RNA, can bind to related miRNAs to further suppress the function of the miRNAs [12, 40]. However, we found WFDC21P could elevate miR-4293 levels via a positive feedback loop. Furthermore, the oncogenic role of miR-4293 was blocked by suppressing WFDC21P. miRNAs have the potential to target hundreds of mRNAs because of imperfect complementarity when binding. Different targets result in different roles of miRNAs [41, 42]. Although WFDC21P did not directly interact with miR-4293, we found that WFDC21P may play a major role in the function of miR-4293, and WFDC21P can be considered as a downstream regulator of the miR-4293 signaling pathway. In the present study, we observed that WFDC21P knockdown almost completely abolished the effects of miR-4293 on proliferation, apoptosis, and metastasis. WFDC21P overexpression could only restore the effects of ASO-4293 suppression to a certain extent, possibly because of DCP2 activation. Transfection of ASO-4293 almost abolished suppression of DCP2 by miR-4293, and DCP2 activation may be greater with ASO-4293 treatment. Even though WFDC21P was overexpressed, its degradation was upregulated by activated DCP2.

STAT3 plays a critical role in many pathophysiological processes, including cancer cell proliferation, anti-apoptosis, and metastasis [43, 44]. We found that STAT3 expression was not only elevated in lung carcinoma, but also positively correlated with the expression of both miR-4293 and WFDC21P. In addition to enhancing STAT3 activation, WFDC21P possibly participates in the regulation of STAT3 signaling [45, 46]. As a regulator of WFDC21P, miR-4293 could further elevate STAT3 phosphorylation. Moreover, by regulating STAT3 activation, WFDC21P could elevate the expression of miR-4293. Hence, STAT3 may play a critical role in the proto-oncogenic capacity of miR-4293 and WFDC21P. STAT3 deactivation may suppress the function of miR-4293 and WFDC21P.

In this study, we found that miR-4293 downregulates the degradation of WFDC21P by directly mediating DCP2, which plays important role in the tumorigenesis of lung carcinoma. Moreover, WFDC21P interference almost completely abolished the effects of miR-4293 on the proliferation of lung carcinoma cells, which indicates that WFDC21P knockdown might be an effective strategy for lung cancer therapy. Our study also demonstrated that miR-4293 and WFDC21P promote tumor growth in vitro and in vivo, which provided a valuable theoretical basis for the discovery of lung carcinoma therapeutic targets and diagnostic markers based on WFDC21P and miR-4293.

## Materials and methods

### Lung carcinoma tissues

Sectioned lung tissues were collected between August 1, 2016, and July 31, 2018, from the Inpatient Department of Medical Oncology, Yantai Shan Hospital, the Teaching Hospital of Binzhou Medical University (Yantai, China). The experiments were performed in accordance with the relevant guidelines of the Code of Ethics of the World Medical Association for experiments involving humans and the Medical Ethics Committee of Binzhou Medical University. Patients provided written informed consent and the study procedures were fully explained before study inclusion. Sixteen patients (8 males and 8 females, aged 31–74 years) who were pathologically diagnosed with NSCLC for the first time and had not yet received chemotherapy, were included in the present study. Fresh NSCLC tissues and controls were obtained from the patients who underwent surgery.

### Real-time qRT-PCR

Real-time qRT-PCR was performed as previously described [47, 48]. Primers used to amplify miR-4293, STAT3, WFDC21P, etc were shown in Supplemental Table 1. qRT-PCR was performed on a Rotor-Gene RG-3000 system (Qiagen, Germany) under the following reaction condition: initial denaturation at 95 °C for 30 s, followed by 40 cycles at 95 °C for 5 s, annealing at 60 °C for 20 s, and extension at 72 °C for 30 s. GAPDH cDNAs served as a positive control.

### Cell lines

The human cell lines (A549, H1299, H1975, and HBE) were obtained from the Shanghai Institute of Cell Biology, China. Cells were cultured in RPMI-1640 medium (Gibco, Grand Island, New York, USA) supplemented with 10% fetal bovine serum (Gibco) in a standard humidified incubator with 5% CO_2_ at 37 °C. The cell lines have been detected without mycoplasma contamination.

### Cell transfection

Cells were seeded in 96-well plates or 6-well plates. Transfection was performed in triplicate at approximately 60% confluence using Lipofectamine TM 2000 (Invitrogen, Carlsbad, CA, USA) according to the manufacturer′s instructions.

### MTT assay

Cell proliferation was measured with an MTT assay as in our previous reports [49, 50]. Cells (1 × 10^4^) in each well of 96-well flat-bottom microtiter plates were treated with microRNAs or plasmids for 48 h. Four hours before the end of incubation, 10 μL MTT (Sigma, St Louis, MO, USA, 5 mg/mL) was added to each well. The supernatant was then removed and 100 μL DMSO (Sigma) was added and the optical density (OD) was measured (570 nm) using an ELISA reader (Multiskan FC, Thermo Fisher Scientific, Boston, MA, USA).

### Apoptosis assays

Apoptotic cells were detected using an Annexin V-FITC/PI kit (KeyGEN Biotech. Co. Ltd., Nanjing, China) according to the manufacturer′s instructions. A total of 1 × 10^4^ cells was collected and analyzed by flow cytometry as previously described [47, 48].

### Western blot

Western blot analysis was performed as described previously. The antibodies used were as follows: rabbit anti-human DCP2 (1:1000; ab28658; abcam, Ltd), rabbit anti-human STAT3 (1:500; bsm-33218M; Bioss, Ltd), rabbit antihuman p-STAT3 (1:800; AP0248), mouse anti-human BIRC3 (1:500; MB0129), rabbit anti-human Bax (1:500; BS6420), Bcl2 (1:500; BS70205), rabbit anti-human Caspase 3 (1:1000; BS61583), rabbit anti-human Caspase 8(1:1000; AP0258), rabbit anti-human Caspase 9 (1:1000; AP0359), and rabbit anti-human GAPDH (1:3000; MB001) all from Bioworld Technology, Ltd).

### Transwell migration assay

Transwell migration assays were performed using Corning Costar Transwell chambers with 8 μm pore size membranes (Sigma, St Louis, MO, USA). After transfection, cancer cells incubated in a 500 μL serum-free medium were seeded into the upper chamber. The lower chamber was filled with 600 μL 1640 medium supplemented with 10% calf serum. After 24 h, cells on the upper surface of the membrane were removed with cotton swabs. Lower chamber cells were stained with 1% crystal violet (Sigma) in 2% ethanol for 20 min. Excess crystal violet was removed by quickly immersing the insert in ddH_2_O for 3–4 s. Stained cells were counted under a microscope (DM6000B, Leica). The assay was repeated three times for each group.

### Colony formation

After transfection in 6 wells plates, cancer cells were incubated for 48 h. Then, transfected cells were harvested, and 1.5 × 10^3^ cells per treatment group were seeded into 6 cm plates for colony formation. On day 12, colonies were stained with 0.25% crystal violet. Colonies were countered and reported as the number ± SEM.

### RNA immunoprecipitation

RNA immunoprecipitation (RIP) experiments were performed using a Magna RIP RNA-Binding Protein Immunoprecipitation Kit (Merk Millipore, Darmstadt, Germany) according to the manufacturer′s instructions. Antibodies for RIP assays against DCP2 were purchased from Abcam-Trading Company (Shanghai, China).

### mRNA transcriptome library preparation and sequencing

Total RNAs from 3 NSCLC and control para-carcinoma tissues were isolated. The preparation of mRNA transcriptome libraries and deep sequencing were performed by Novogene Bioinformatics Technology Cooperation (Beijing, China). mRNA was gathered and strand-specific sequencing libraries were generated following the manufacture’s recommendations. RNA-Seq was performed on an Illumina Hiseq 2000 platform and 100 bp paired-end reads were generated according to Illumina’s protocol.

### RNA-Seq data analysis

The adapter sequences were removed from the raw sequencing data and the individual libraries were converted to the FASTQ format. Sequence reads were aligned to the human genome (hg19) with TopHat2 and the resulting alignment files were reconstructed by Cufflinks50 and Scripture51. For mRNA analyses, the RefSeq database (Build 37.3) was chosen as the annotation reference.

The read counts of each transcript were normalized to the length of the individual transcript and to the total mapped fragment counts in each sample and expressed as fragments per kilo-base of exon per million fragments mapped (FPKM) in each sample. The mRNA differential expression analyses for carcinoma versus para-carcinoma (CA vs. PARA). An adjusted *P* value < 0.05 (Student’s *t*-test with Benjamini–Hochberg false discovery rate (FDR) adjustment) was used as the cut-off for significantly differentially expressed genes. Differentially expressed genes were analyzed by enrichment analyses to detect over-represented functional terms present in the genomic background.

### Continuously monitor cell migration

To continuously monitor cancer cell migration, 1 × 10^4^ cells per well were seeded into the top chamber of a CIM plate, which was then incubated on the RTCA station (xCELLigence System, Roche, Mannheim, Germany). Changes in cell migration were monitored in real-time.

### Xenografts in mice

A549 cells stably expressed WFDC21P, or untreated A549 cells (5 × 10^6^) in 0.1 mL PBS, transfected with miR-4293, WFDC21P-small interfering RNAs (siRNAs) WFDC21P were transplanted subcutaneously into the right or left flanks of 5–6-week-old male BALB/c-nu/nu mice weighing 18–20 g (Charles River. Beijing, China). Animals were grouped by simple randomization using a random number table. The principle of sample sizing has followed the “The ARRIVE Guidelines” [51]. The maximum and minimum tumor diameters were measured every 3 d. The second time of injection of the xenografts with siRNA or miRNA (Supplemental Table 2) was performed on the 14th day after the first time treatment. The mice were house in a specific-pathogen-free (SPF) environment. Tumor volume (mm^3^) = A × B^2^/2, where A and B are the maximum and minimum tumor diameters, respectively. After 4 weeks, nu mice were given euthanasia by amobarbital injection of 3 times standard does. The tumors were anatomized and weighed. The investigator had no bias and special tendency in the processing of animal experiments. All animal experiments were approved by the Committee on the Ethics of Animal Experiments of Binzhou Medical University and performed in accordance with the National Institutes of Health guide for the care and use of laboratory animals.

### Statistics

SPSS 22.0 software (IBM Corp., Armonk, NY, USA) was used to analyze statistical significance. Normally distributed data were expressed as mean ± SD, and Student’s *t*-test was used to compare two averages and ANOVA was used for mean comparison of multiple groups. If the assumption of homogeneity of variance is accepted or rejected, the LSD test or Games–Howell test is used to compare the mean of different samples. Abnormally distributed data were expressed as median (interquartile range), the Mann–Whitney U test was used to compare two groups, and Kruskal–Wallis H test was used to compare multiple groups. Pearson’s correlation was used to analyze the association between different variables. *p* < 0.05 is considered statistically significant differences.

## Supplementary information

Supplemental data

## Data Availability

All data generated during this study are included either in an article or in the additional files.
