# Supplementary material for: miR-4293 upregulates lncRNA WFDC21P by suppressing mRNA-decapping enzyme 2 to promote lung carcinoma proliferation
Source: Cell Death Dis. 2021 Jul 23;12(8):735. doi: 10.1038/s41419-021-04021-y (PMC8302752; doi:10.1038/s41419-021-04021-y)
Supplement: Supplementary file 1 — Supplemental data [file 41419_2021_4021_MOESM1_ESM.docx]

**Supplemental Data**

**miR-4293 upregulates lncRNA WFDC21P by directly suppressing mRNA-decapping enzyme 2 to promote lung carcinoma proliferation**

**1. Table S1.** Primers

| **Primers** | **Sequence** |
| --- | --- |
| miR-4293 | **Forward：**5′- ACCAGCCTGACAGGAACAG -3′  **Reverse：**5′- CGAACATGTACAGTCCATGGATAG - 3′ |
| WFDC21P | **Forward：**5′-CCAAGACCTGAGCCCTGTAA-3′  **Reverse：**5′-ATAGAGGTGGCTGTCTGATGCT -3′ |
| STAT3 | **Forward：**5′- TGCGGAGAAGCATCGTGAGT-3′  **Reverse：**5′- CCTCCAATGCAGGCAATCTGT-3′ |
| Human 5S rRNA | **Forward：** 5’-GCCATACCACCCTGAACG-3’  **Reverse：** 5’-CGAACATGTACAGTCCATGGATAG-3’ |
| DCP2 | **Forward：**5’ -CTGTTCCTGCTGTGGGTCCTC-3’  **Reverse：**5’-ACATTGCCTCCCTGCATTTC-3’ |
| GAPDH | **Forward：**5’ -GTCTTCACCACCATGGAGAAGG-3’  **Reverse：**5’- GCCTGCTTCACCACCTTCTTGA-3’ |

**2. Table S2.** miRNA, ASO and siRNA sequences

| **microRNA** | **Sequence** |
| --- | --- |
| miR-4293 | 5’-CAGCCUGACAGGAACAG-3’ |
| Mu-4293 | 5’-CUAUCUGACAGGAACAG-3’ |
| ASO-4293 | 5’- CUGUUCCUGUCAGGCUG-3’ |
| Si-DCP2-1 | 5’- ACUCGGAUUGCAUUGUCUC -3’ |
| Si-DCP2-2 | 5’- AUAUGUUGGUACUCCCAUU -3’ |
| Si-WFDC21P | 5’- GAGUUAUCUUAAGGAUCAU -3’ |

**
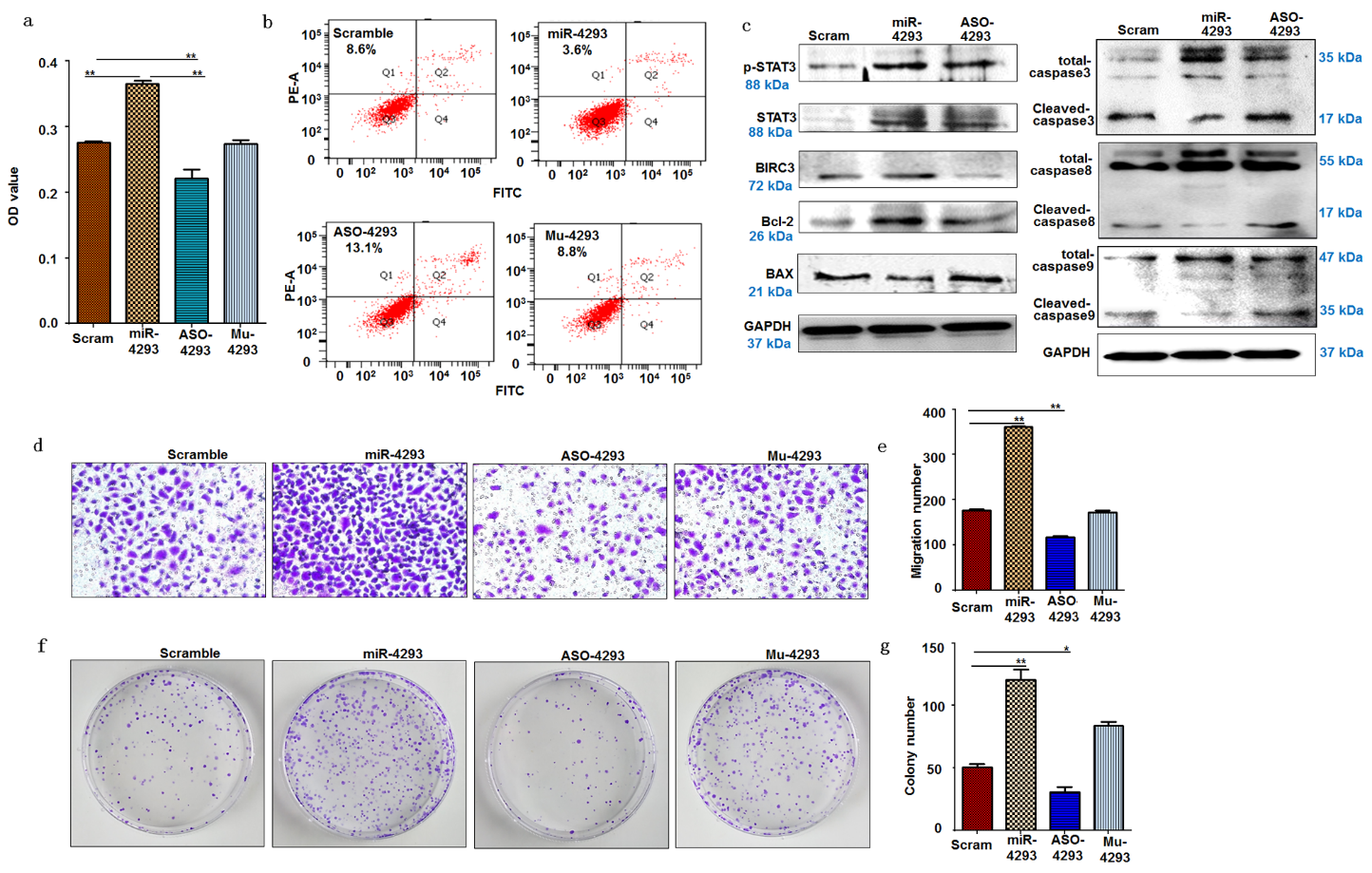
3. Figure S1.**

**Supplemental Fig.1 miR-4293 regulates H1975 cell proliferation, migration and apoptosis.** (**a**) MTT assay detected at 24 h post-transfection. miR-4293 significantly promoted, while ASO-4293 inhibited H1975 cell growth (***P*<0.01) compared with mu-4293 or scramble control. (**b**) ASO-4293 could effectively induce H1975 cell apoptosis compared with miR-4293, mu-4293 or scramble control. (**c**) Western blot detection for STAT3 and apoptosis-related factors (Bcl-2, Bax, BIRC3, and Caspase 3,8,9) in miR-4293-, or ASO-4293-, or mu-4293-treated H1975 cells. (**d, e**) Transwell migration assay. ASO-4293 suppressed cell migration compared with mu-4293 or scramble control (***P*<0.01). (**f, g**) Colony formation assays showed that that miR-4293 significantly increase survival of H1975 seeded sparsely (***P*<0.01), but ASO-4293 suppressed colony formation (***P*<0.01). Data are presented as mean ± s.d.

**4. Figure S2.**

**
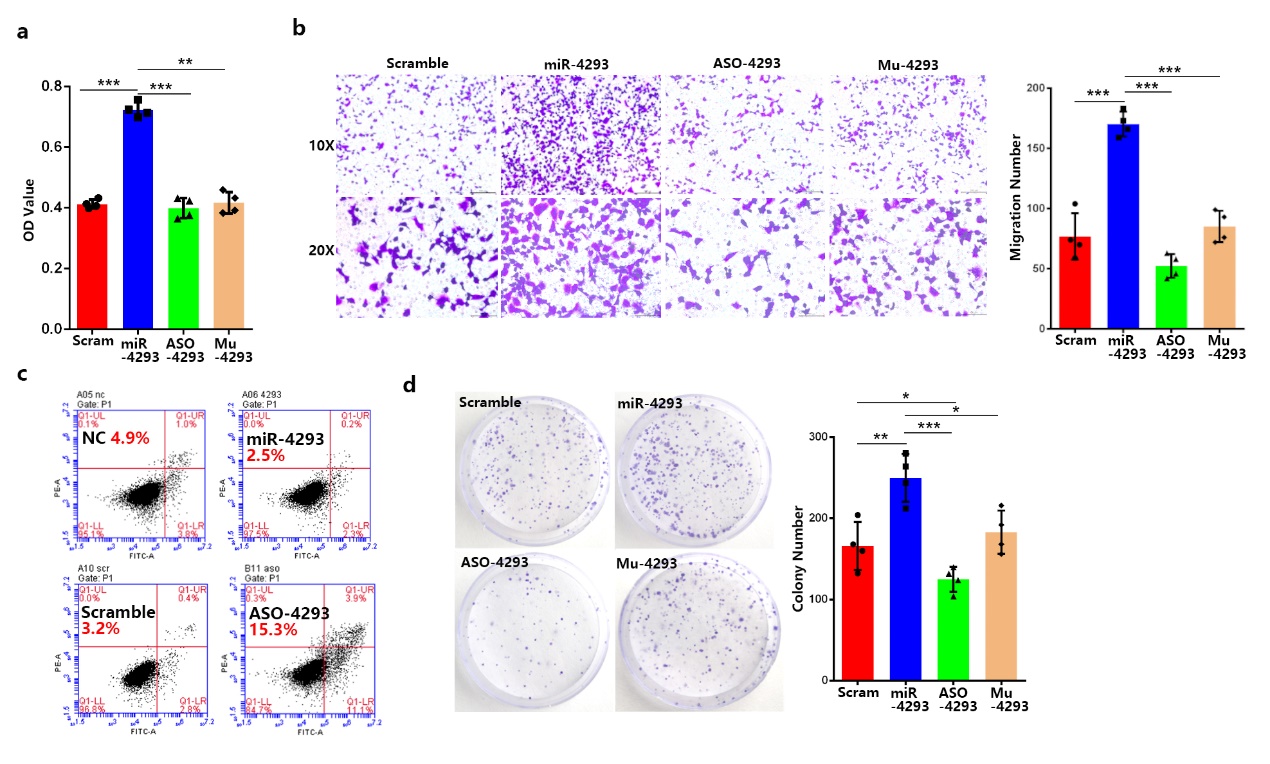
**

**Figure S2: miR-4293 promoted proliferation and migration of H1299.** **a** MTT assay of H1299 at 24 h post-transfection of miR-4293, ASO-4293, mu-4293 or scrambled. Data are expressed as mean ± SD. ** p<0.01, *** p<0.001; ANOVA test. **b** Transwell migration assay of H1299 at 24 h post-transfection of miR-4293, ASO-4293, mu-4293 or scrambled. Data are expressed as mean ± SD. * p<0.05, *** p<0.001; ANOVA test. **c** FACS analysis of H1299 cell apoptosis at 24 h post-transfection of NC, miR-4293, scramble and ASO-4293. **d** Colony formation assays of H1299 at 24 h post-transfection of miR-4293, ASO-4293, mu-4293 or scrambled. Data are expressed as mean ± SD. * p<0.05, ** p<0.01, *** p<0.001; ANOVA test.

**5. Figure S3.**

**
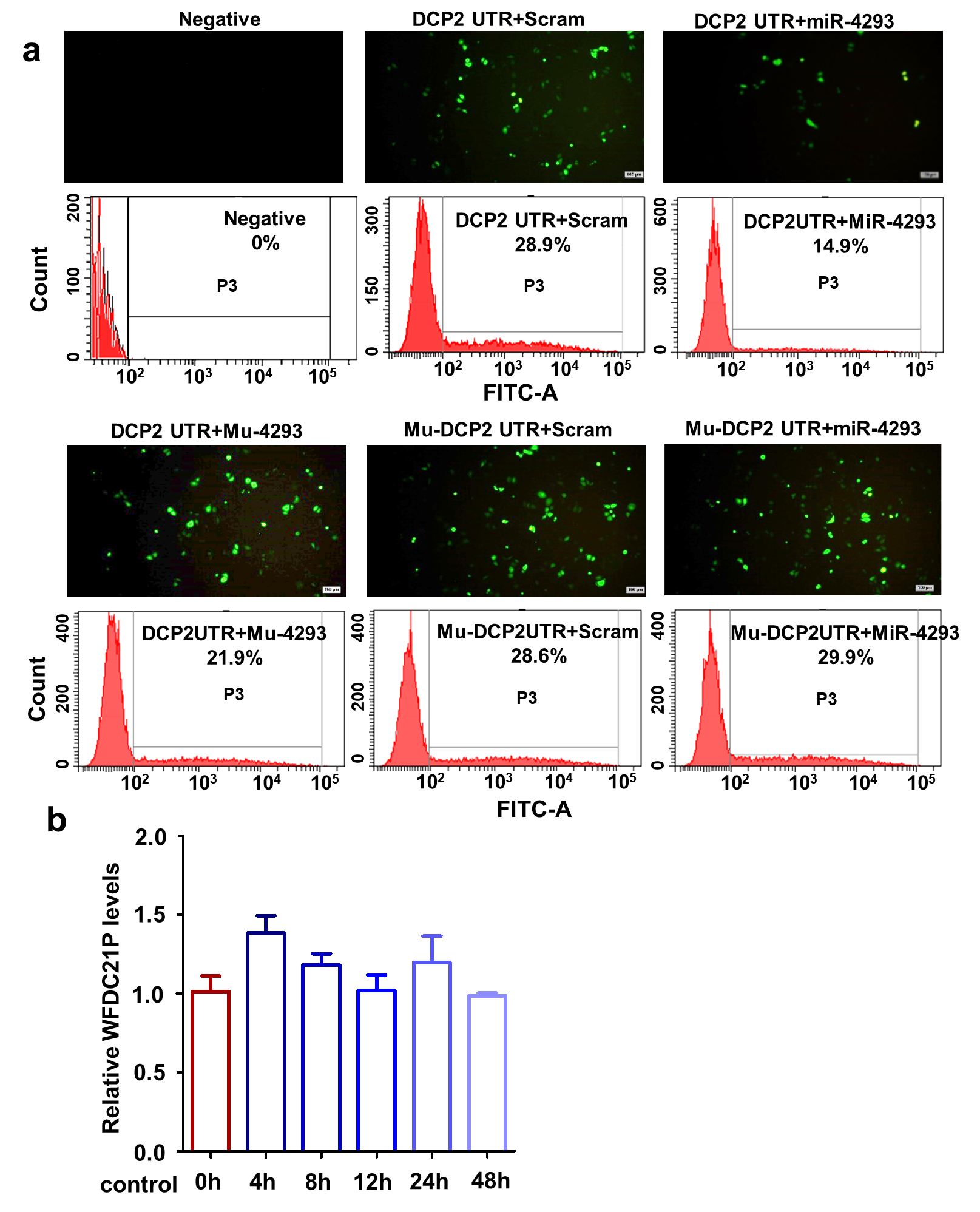
**

**Supplemental Fig.3 a** The effect of miR-4293 on regulating GFP reporter plasmid containing DCP2-3′UTR**.** Upper panel, fluorescence analysis (bar = 100 μM); lower panel, FACS analysis. **b** qRT-PCR analysis of WFDC21P level in 0h, 4h, 8h, 12h, 24h, 48h after transfection of pcDNA3.1. Data are expressed as mean ± SD for triplicate experiments. ** p<0.01; ANOVA test.

**6. Figure S4.**

a

b

**
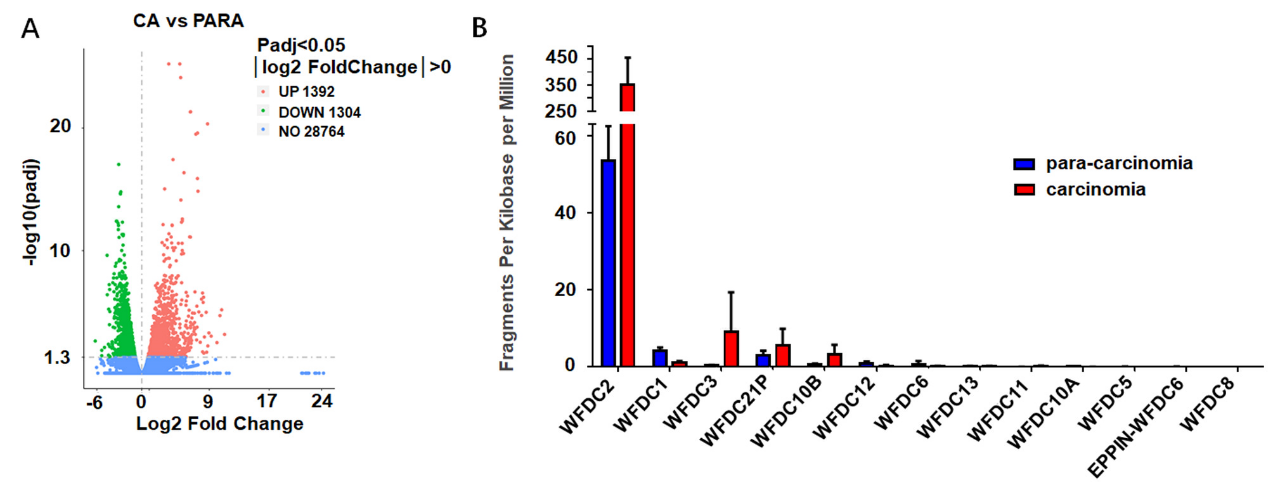
**

**Supplemental Fig. 4 RNA-sequencing detected the expression of WFDC family members. a** Vocalno Plot indicated up and down regulated mRNAs in lung carcinoma tissues compared with para-carcinoma tissue. **b** FPKM analysis from RNA-seq dataset showed the expression of WFDC family members in carcinoma tissues compared with para-carcinoma tissue.

**
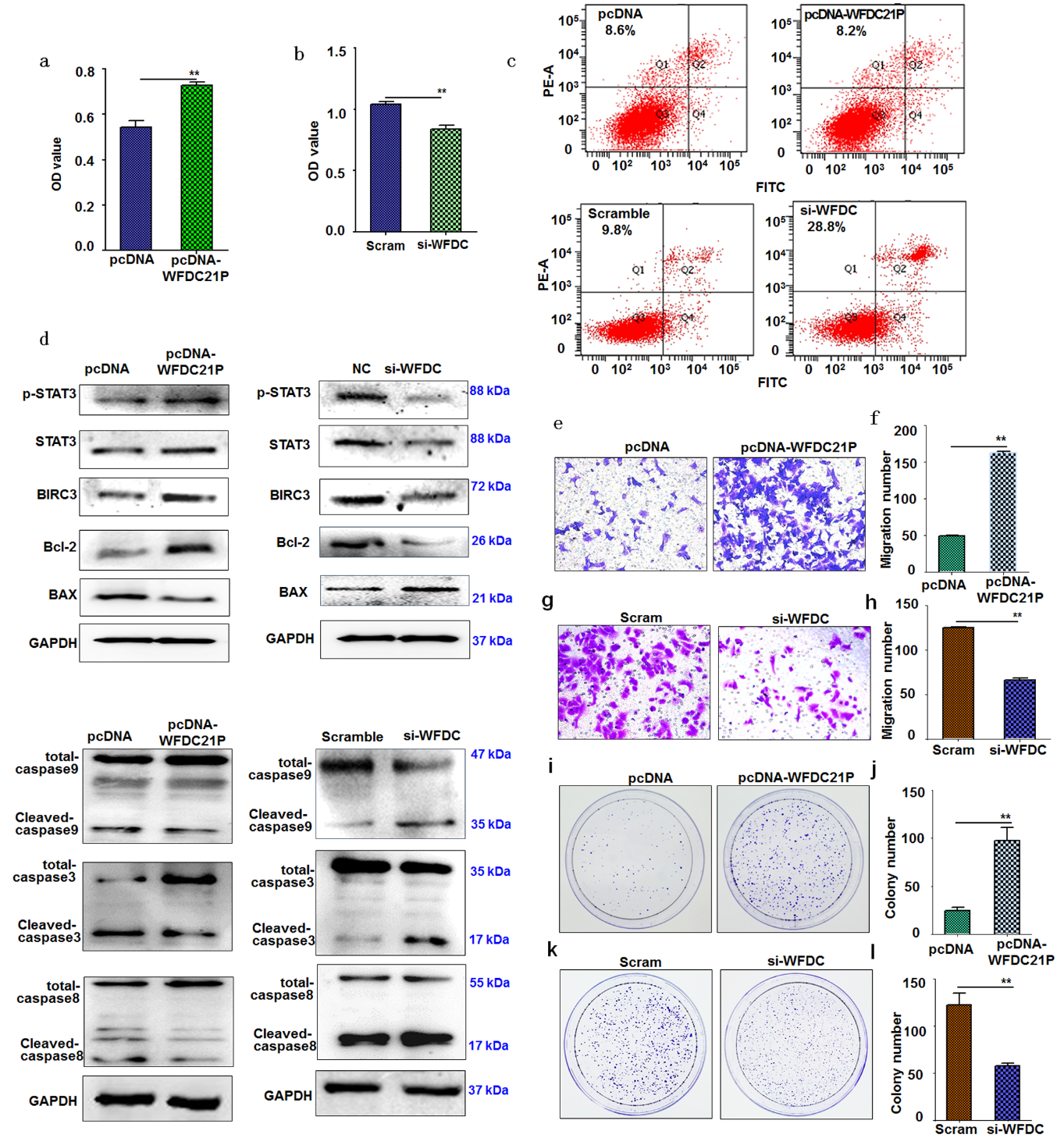
7. Figure S5.**

**Supplemental Fig. 5 WFDC21P regulates H1975 cell proliferation, migration and apoptosis. a, b** MTT assay. The above-mentioned results showed that pcDNA-WFDC21P transfection could significantly elevate the expression of WFDC21P, and Si-WFDC transfection led to knocking down WFDC21P levels. Then, our results showed that overexpressing WFDC21P promoted H1975 cell proliferation (a, ***P*<0.01), while interfering WFDC21P inhibited H1975 cell growth (b, ***P*<0.01). **c** FACS. Much more apopotic cells were found in si-WFDC21P-, but not in pcDNA-WFDC21P-treated H1975 cells compared with control. **d** Western blot. The expression of p-STAT3, BRIC3, and Bcl-2 increased, but BAX and cleaved-caspase 3,8,9 decreased in pcDNA-WFDC21P-treated H1975 cells. si-WFDC21P treatment resulted in lower levels of p-STAT3, BRIC3, and Bcl-2, and higher levels of BAX and cleaved-caspase 3,8,9. **e-h** Transwell assay. Overexpressing WFDC21P promoted H1975 cell migration (e, f, ***P*<0.01), while interfereing WFDC21P leaded to suppressing H1975 migration (g, h, ***P*<0.01). **i-l** Colony formation. The colony number increased in pcDNA-WFDC21P-treated H1975 cells (i, j, ***P*<0.01), while decreased in interfereing WFDC21P-treated cultures (k, l, ***P*<0.01). Data are presented as mean ± s.d.

**8. Figure S6**


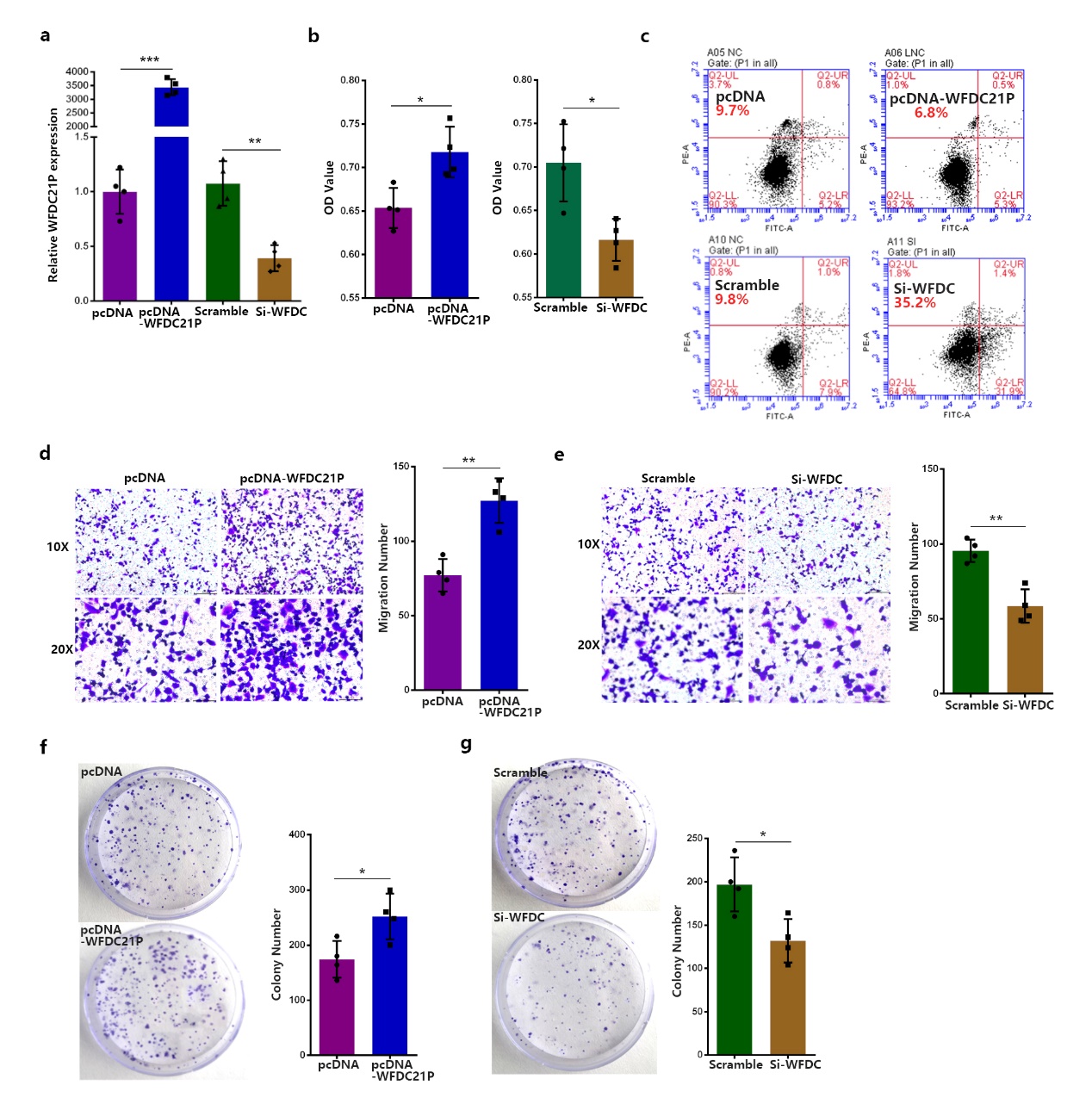


**Figure S6: WFDC21P promoted proliferation, migration but suppressed apoptosis of H1299.** **a** qRT-PCR analysis of WFDC21P level in H1299 cells with transfection of NC, miR-4293 mimic, scramble or ASO-4293. Data are expressed as mean ± SD. ** p<0.01, *** p<0.001; ANOVA test. **b** MTT assays. The abovementioned results showed that pcDNA-WFDC21P transfection elevated WFDC21P levels, and Si-WFDC transfection knocked down WFDC21P levels. Then, MTT assays of H1299 at 24 h post-transfection of pcDNA-WFDC21P and pc-DNA (left panel), scramble and si-WFDC (right panel) to further analyze the role of WFDC21P in regulating cell growth. Data are expressed as mean ± SD. * p<0.05; Student’s t test. **c** FACS analysis of H1299 cell apoptosis at 24 h post-transfection of pc-DNA, pcDNA-WFDC21P, si-WFDC21P and scrambled control. **d-e** Transwell assays of H1299 migration. (d) pcDNA vs pcDNA-WFDC21P; (e) scramble vs si-WFDC. Data are expressed as mean ± SD. ** p<0.01; Student’s t test. **f-g** Colony formation assays of A549. (f) pcDNA vs pcDNA-WFDC21P; (g) scramble vs si-WFDC. Data are expressed as mean ± SD. * p<0.05; Student’s t test.

**
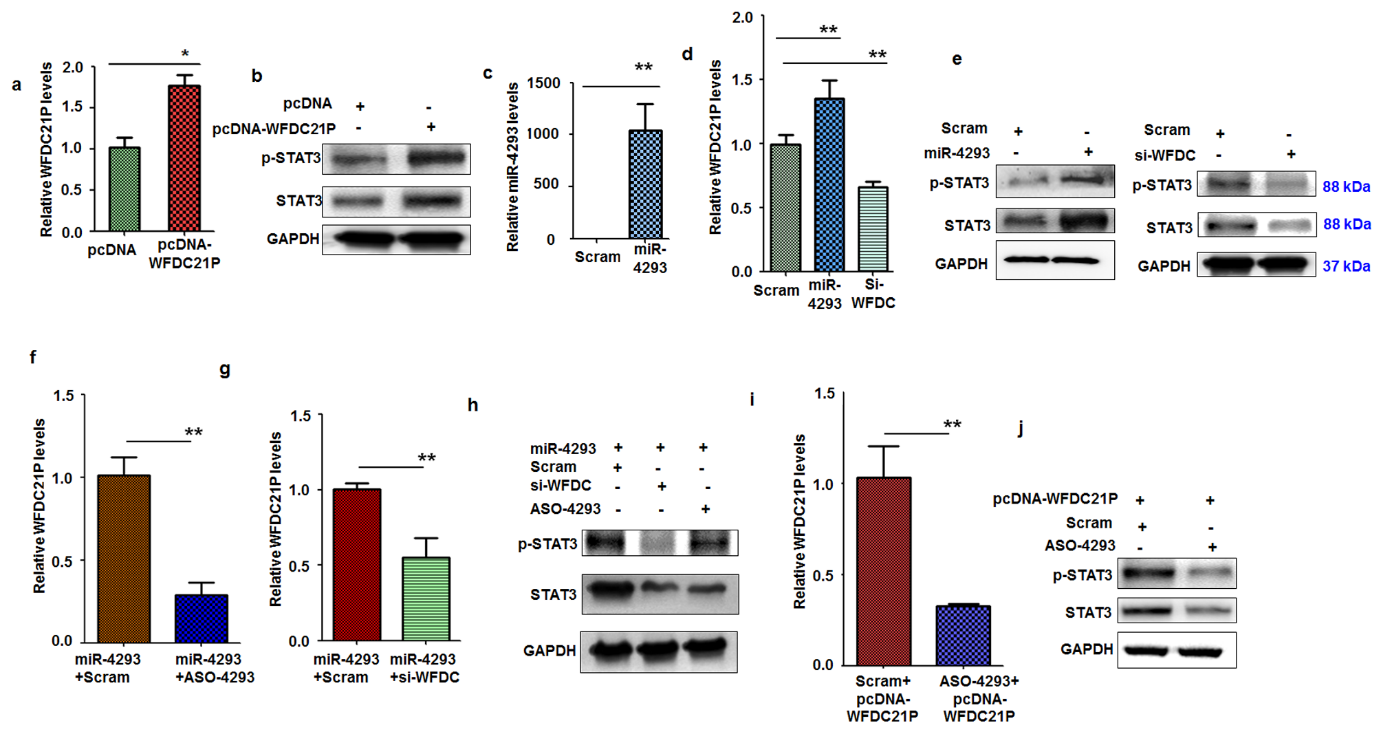
9. Figure S7.**

**Supplemental Fig. 7 Detection miR-4293, WFDC21P and STAT3 expression in tumor xenografts**

(**a**) qRT-PCR analysis. WFDC21P was significantly over-expressed in pcDNA-WFDC21P-treated xenografts compared with control (**P*<0.05). (**b**) Western blot. STAT3 phosphorylation increased in pcDNA-WFDC21P-treated xenografts compared with control. (**c**) qRT-PCR analysis. miR-4293 expression increased in miR-4293-treated xenografts compared with control treatment (***P*<0.01). (**d**) qRT-PCR analysis. WFDC21P expression increased in miR-4293-treated xenografts, but decreased in si-WFDC21P-treated xenografts compared with scram control (***P*<0.01). (**e**) Western blot. The levels of STAT3 and its phosphorylation increased miR-4293-treated xenografts, and decreased in si-WFDC21P-treated xenografts. (**f**) qRT-PCR analysis. ASO-4293 could effectively inhibit miR-4293 expression in xenografts compared with control (***P*<0.01). (**g**) qRT-PCR analysis. WFDC21P expression decreased in miR-4293+si-WFDC21P-treated xenografts compared with miR-4293+scram treatment control (***P*<0.01). (**h**) Western blot. STAT3 phosphorylation decreased in miR-4293+si-WFDC21P-treated xenografts or miR-4293+ASO-4293-treated xenografts. (**i**) qRT-PCR analysis. WFDC21P expression decreased in pcDNA-WFDC21P+ASO-4293-treated groups compared with pcDNA-WFDC21P+control treatment in xenografts (***P*<0.01). (**j**) Western blot. The levels of STAT3 and its phosphorylation decreased in pcDNA-WFDC21P+ ASO-4293-treated groups compared with pcDNA-WFDC21P+control treatment (***P*<0.01).
